# A Video-Based Framework for Automatic 3D Localization of Multiple Basketball Players: A Combinatorial Optimization Approach

**DOI:** 10.3389/fbioe.2020.00286

**Published:** 2020-04-30

**Authors:** Lucas Antônio Monezi, Anderson Calderani Junior, Luciano Allegretti Mercadante, Leonardo Tomazeli Duarte, Milton S. Misuta

**Affiliations:** ^1^Faculty of Physical Education, University of Campinas, Campinas, Brazil; ^2^School of Applied Science, University of Campinas, Limeira, Brazil

**Keywords:** machine learning, sports, computer vision, player detection, non-invasive method, tracking

## Abstract

Sports complexity must be investigated at competitions; therefore, non-invasive methods are essential. In this context, computer vision, image processing, and machine learning techniques can be useful in designing a non-invasive system for data acquisition that identifies players’ positions in official basketball matches. Here, we propose and evaluate a novel video-based framework to perform automatic 3D localization of multiple basketball players. The introduced framework comprises two parts. The first stage is player detection, which aims to identify players’ heads at the camera image level. This stage is based on background segmentation and on classification performed by an artificial neural network. The second stage is related to 3D reconstruction of the player positions from the images provided by the different cameras used in the acquisition. This task is tackled by formulating a constrained combinatorial optimization problem that minimizes the re-projection error while maximizing the number of detections in the formulated 3D localization problem.

## Introduction

Recent advances in sports science have been made possible due to the development of appropriate technology. For instance, computer-aided systems can be applied in several sports to obtain both high and low-level data about the performance of a player or team. In basketball, a typical example of low-level data is the position of a player on the court. Knowing the players’ positions reveals important information because it can be used to compute higher-order data related to technical and physical activities as well as tactical analysis. As claimed in the sports science literature (Hopkins et al., 1999; McGarry et al., 2002), sports complexity must be analyzed at competitions, which means that non-invasive methods are preferable for acquiring data such as player position. In this respect, the fields of computer vision, image processing, and machine learning can play a role as they provide useful tools for designing non-invasive video-based systems to collect player motion data during official basketball matches. In the last two decades, researchers have made important contributions to individual and team sports analysis through the development of video-based computer-aided systems (Intille and Bobick, 1995; Iwase and Saito, 2004; Figueroa et al., 2006b; Barros et al., 2007, 2011; Gomez et al., 2014; Morais et al., 2014). In team sports, these studies particularly focus on tracking the players (Figueroa et al., 2006b; Barros et al., 2011; Morais et al., 2014) and the ball (Stennett, 2003; Spagnolo et al., 2013). When tracking objects, one goal is to obtain the object’s trajectory as a function of time, however, doing so requires a previous step: accurately detecting the object of interest. Therefore, tracking by video-based methods necessarily requires both object detection and determination of the object’s location within the scene. This information can then be used to associate the identified objects with their trajectories. The desirable objects to track in team sports applications for evaluating game dynamics are the players, the referees, and the ball. As reported by Figueroa et al. (2006b); Barros et al. (2007, 2011), and Morais et al. (2014), several 2D approaches have been used in video-based applications for player and referee detection, localization, and tracking. In a 2D approach, only two spatial coordinates are taken into account, however, it is also possible to consider three spatial coordinates (a 3D approach), because 3D data provides a huge variety of information that will give coaches support in their training process, besides of the possibility in get game contextualized performance data for both physical and technical aspects. Generally, 3D methods have mainly been considered for ball tracking applications (Ohno et al., 2000; Stennett, 2003; Poliakov et al., 2010). However, the vertical component of player position is essential information in basketball analysis because the players frequently jump during the game. Player detection in basketball is not an easy task; artifacts such as player occlusions, strong shadows cast by players, and sharp reflections from the polished floor significantly affect the segmentation process (Alahi et al., 2009). The midpoint between the feet and the bottom center of a player’s bounding box have both been used as reference points that determine a player’s position within the image, allowing subsequent reconstruction of the player’s 2D position on the court (Iwase and Saito, 2004; Figueroa et al., 2006b; Lu et al., 2009; Barros et al., 2011). However, the use of the midpoint between the feet as a reference point can lead to problems during the segmentation stage, especially when the legs have a color pattern similar to the basketball court itself. Because a low error rate in identifying players’ positions is key for tracking algorithms, other reference points must be explored. One possible candidate in this respect is the player’s head, which is less affected by the aforementioned artifacts. In fact, player head shape, color, and size provide more stable and invariant features than feet. Moreover, choosing head position on the court space as a reference point for locating a player and detecting heads in multiple cameras are measures that conform well to a 3D reconstruction approach. Bearing in mind the limitations and requirements discussed above, in this paper, we propose a video-based framework for automatic 3D localization of multiple basketball players. The paper is organized as follows. Section named Proposed Framework presents the two stages of our method: player detection and 3D reconstruction. Then, in Framework Performance Evaluation Section, we provide a set of numerical experiments to assess the performance of the proposed method. Discussion Section contains a talk of the results, and Conclusion Section presents the closing considerations. Approval for video data collection was obtained from the Brazilian National Basketball League and Limeira Basketball Association.

## Materials and Methods

### Proposed Framework

**FIGURE 1:**
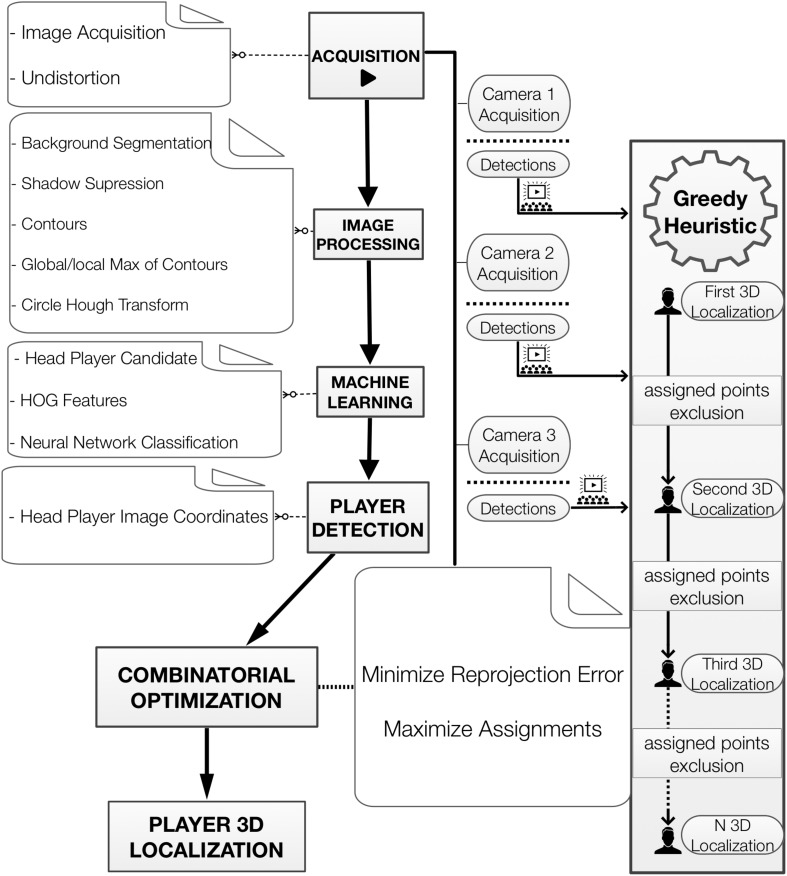
Framework Diagram. The key tasks for player detection and 3D localization. For better understanding the structure of the method we produced a series of videos of the main process used: S1-detection, S2-1st round optimization, S3-2nd round optimization, and S4-3rd round optimization.

The proposed framework, summarized in Figure 1, comprises two main parts. The first part addresses the identification of the players’ heads at the camera image level. As will be detailed in section, player detection is conducted after image acquisition and requires image processing and machine learning procedures. The second stage of our proposal concerns the 3D reconstruction of player positions. This task, as will be discussed in section, can be addressed by formulating a combinatorial optimization problem.

### Player Detection

#### Acquisition

Image acquisition is the first step required for player detection (Figure 1). To accomplish this, a dedicated capturing program was built (using Vimba SDK, OpenCV, and C/C++) to directly record and synchronize videos from multiple camera views to a computer. The video data (1,038 × 7,765 Hz) used in this work were acquired using three static industrial FireWire cameras (Allied Vision Technologies GmbH, with 6 mm lens) attached inside protected cages at the highest possible places in the gym (12 m from the ground). To extend the FireWire connection for the cameras and achieve the right locations for framing the court, a converter adapter was plugged into each camera using optical fiber (Gefen Firewire 1394 400/800 Extender). Because these cameras used aspherical lenses (C-mount, 6 mm), it was necessary to perform a distortion correction over the entire image. The correction protocol involved a chessboard (planar pattern) which was moved so the cameras could take images at different orientations. In this manner, a closed-form solution was obtained and refined for modeling the radial distortion (Zhang, 2000).

#### Image Processing

**FIGURE 2:**
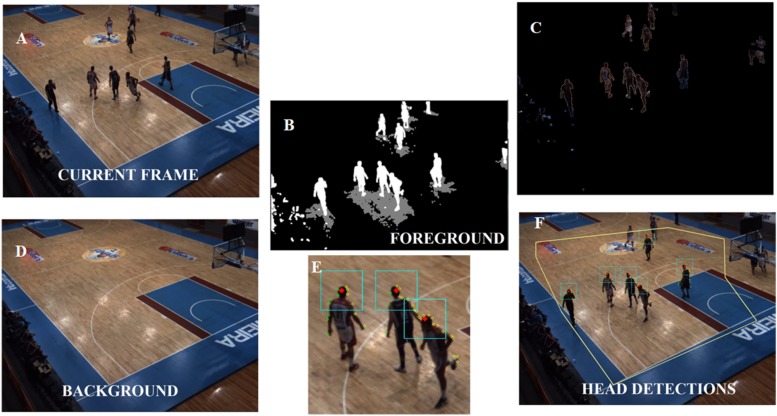
Process inside of Player Detection stage. Player detection process over a delimited interest area **(E)** with some steps depicted. The video image **(A)** is used to model the static background image of the court **(B)**, and the foreground mask **(C)** is the result of subtracting the background **(D)**. The image in **(F)** is a closer view of the three player- head candidates.

In order to illustrate part of the player detection process, we provide in Supplementary Video S1. The first step in player detection is based on segmentation, which separates parts of the image. For sports applications, these parts can be the court or playing field, the players, and balls or other gear. Some image processing tools were used to perform the segmentation of the basketball players. The basic idea was to separate static image regions from moving regions and then perform background segmentation to extract the portions of the images that showed the basketball court while keeping the parts that showed the players (Figure 2).

The Gaussian Mixture-based Background/Foreground Segmentation algorithm was used to do this (Zivkovic, 2004). With the background model, we can also detect shadows and re-mark any shadow pixels marked with foreground labels to background labels (Prati et al., 2003). Finally, noise suppression using image processing techniques that rely on morphological filtering (erosion followed by dilation) was applied to the foreground-mask (Figueroa et al., 2006a).

#### Machine Learning

**FIGURE 3:**
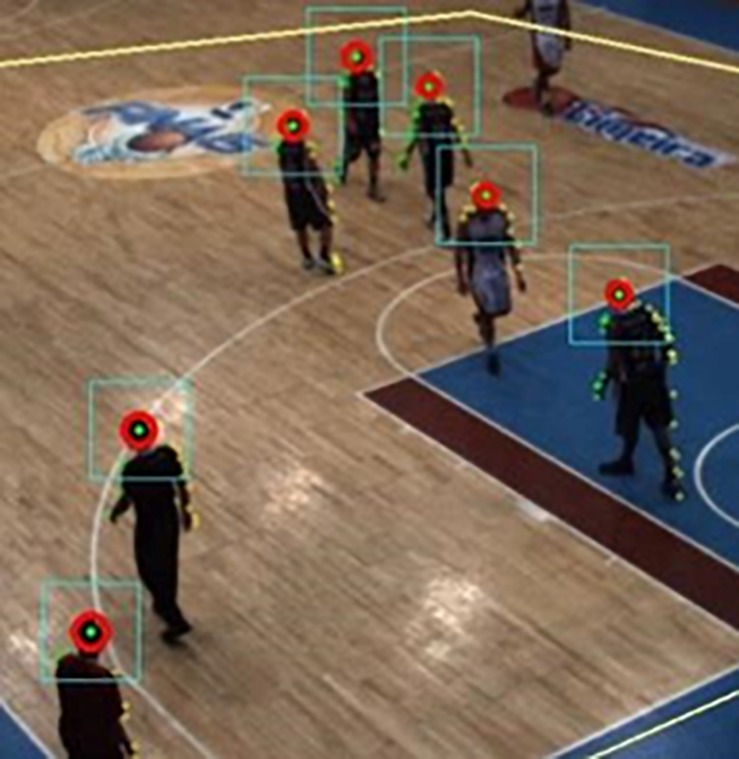
Head candidates’ points. Heads enclosed by the Circle Hough Transform (the square around of the heads delimited region for a computation of HOG features).

The next step is to identify the players’ heads. To do that, the first task is to estimate the contour of a player in the binary foreground image (Suzuki and Be, 1985). Due to the high number of players in the area, the contours found contained frequently more than one player. The highest point in a contour curve may be directly related to a player’s head if the contour contains just one player (Figure 2, f, player from left), however, when the contour curve encloses multiple players (Figure 2, f, two players from right), the highest point can identify only one player’s head. Therefore, to circumvent this issue, the location of the global maximum as well as of the local maxima are taken into account to search for circular patterns related to players’ heads. Thus, a circle was fitted in the grayscale foreground mask near the places of maxima. Then, the Circle Hough Transform (Yuen et al., 1990) was adopted to obtain the best circle (taking head size into account) that was not too far away from the original local maxima (Figure 3).

**FIGURE 4:**
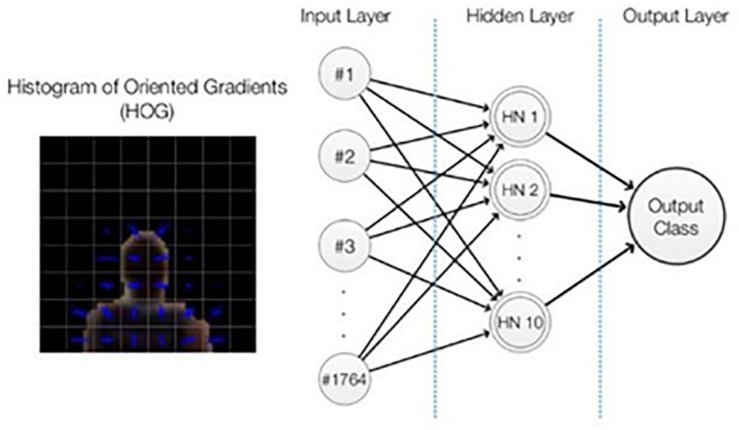
Neural network and histogram of oriented gradients representations. Architecture of neural network adopted with the input variables selected.

A classification into \head” or \non-head” is the ultimate goal of player detection; therefore, the candidate points were classified by a multilayer perceptron neural network, which had previously been trained. The features used were the Histogram of Oriented Gradients, HOG (Dalal and Triggs, 2005) using a fixed square region around the candidate player point (Figures 3, 4).

**FIGURE 5:**
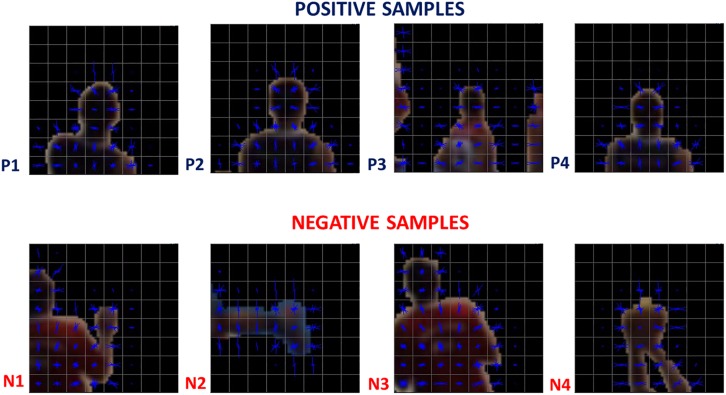
Training samples examples. HOG features of positive and negative samples used as inputs for the neural network.

The centers of the circles obtained are considered as the candidate points, however, these points are not necessarily heads – they could represent a raised arm (Figure 5, N1), the ball (Figure 5, N4), or any other non-head body segment. The candidate points are analyzed only if they appear inside of the interest area (a pre-determined polygon, Figure 2) thus, the high image variability caused by spectators and objects in areas beyond the court does not affect the detection process.

The chosen architecture was a multilayer perceptron feed-forward network with 10 hidden neurons in one hidden layer. The neural network was trained with a back-propagation algorithm (Rumelhart et al., 1986). To train and test the classifier, briefly, we selected a total of 30,009 labeled samples (Figure 5) and analyzed whether the HOG features and neural network classifications were suitable. The samples, collected from an official game (Game 1), were divided into three subsets: training (70%), validation (15%), and test (15%). Finally, reproducibility was checked using a different game (Game 2, 2,027 samples) with samples acquired in a scenario in which the players were wearing different jerseys (the visiting team) and players who had never appeared in the previous dataset of 30,009 samples. The neural network input layer had 1,764 neurons (Figure 4), which correspond to the values from the HOG features (Dalal and Triggs, 2005).

### Reconstruction

**FIGURE 6:**
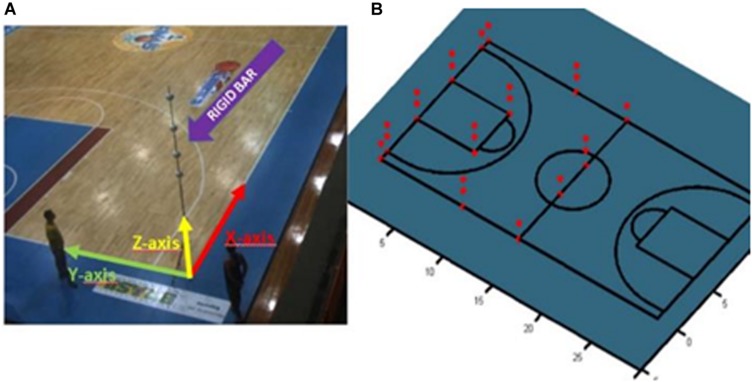
Camera Calibration. **(A)** Rigid bar used for camera calibration, the adopted court reference system, and the calibration points (red, **B**) that appeared at least twice in the camera images.

A prerequisite task for a 3D reconstruction of a given point is to calibrate the cameras. The camera calibration aimed to estimate the parameters of each camera so we could later transform the image coordinates of the player reference point (the head in this paper) to the global coordinates associated with the court dimensions. After correcting for image distortion, a direct linear transform, DLT (Abdel-Aziz et al., 2015), was adopted to perform 3D camera calibration and player reconstruction (Qingchao et al., 1996; Rossi et al., 2015). In this calibration procedure, the intersections of the lines on the basketball playing court were chosen as reference spots; the measurements for these intersections (2D positions) were obtained from the official FIBA rules manual. The origin of the global system was defined at the intersection of one of the lateral lines (X-axis) with one of the bottom lines (Y-axis). Therefore, for any spot selected on the court plane, we placed a rigid vertically oriented bar (checked with a spirit level) that had demarcations along its length showing known measurements. At these demarcation points, white Styrofoam balls (with diameters of 15 cm) were fixed so they would be easy to visualize in camera images (Figure 6).

By measuring some points in the image with known real coordinates, it then becomes possible to solve for any point in the system using (Equations 1 and 2) to estimate the eleven DLT parameters. Eleven unknown variables require eleven or more equations, which means that a minimum of six pairs of points between the image 155 and the real measurements were required (because a point pair provides two equations) (Wood and Marshall, 1986; Abdel-Aziz et al., 2015). The parameters A1 to A11 are associated to the relation between object in space (position X, Y, and Z) and its image in the plane (position x and y). The variable k denote to the camera index. For further details about DLT, please verify Abdel-Aziz et al. (2015) and http://www.kwon3d.com/theory/dlt/dlt.html. The absolute re-projection errors of the calibration points that appeared at least twice (36 points, Figure 6B) averaged 0.026 m (X-axis), 0.031 m (Y-axis), and 0.043 m (Z-axis). The DLT equations are as follows:

(1) $$\left(\lambda_{1_{k}}-\lambda_{3_{k}}\,x_{p_{k}}\right)\,\text{X}+\,\left(\lambda_{4_{k}}-\lambda_{6_{k}}\,x_{p_{k}}\right)\,\text{Y}+\,\left(\lambda_{7_{k}}-\lambda_{9_{k}}\,x_{p_{k}}\right)\,\text{Z}\;+\lambda_{10_{k}}-x_{p_{k}}\,=\,0\,$$

(2) $$\left(\lambda_{2_{k}}-\lambda_{3_{k}}\,y_{p_{k}}\right)\,\text{X}+\,\left(\lambda_{5_{k}}-\lambda_{6_{k}}\,y_{p_{k}}\right)\,\text{Y}+\,\left(\lambda_{8_{k}}-\lambda_{9_{k}}\,x_{p_{k}}\right)\,\text{Z}\;+\lambda_{11_{k}}-y_{p_{k}}\,=\,0\,$$

After obtaining the parameters, it was then possible to reconstruct the X, Y, and Z coordinates in court space from at least two pairs of camera image coordinates.

The goal of the proposed 3D reconstruction process is to estimate the 3D localization of all the players in the scene. The process is built upon a constrained combinatorial optimization problem. The underlying problem-to assign the points detected to a true player-can be modeled by the following assignment matrix:

(3) $$A_{m,n}=\left(\begin{array}{c} a_{1,1}\,a_{1,2}\,\cdots \,a_{1,n}\\ a_{2,1}\,a_{2,2}\,\cdots \,a_{2,n}\\ \begin{array}{cc} \vdots & \vdots \end{array}\;\begin{array}{cc} \ddots & \vdots \end{array}\\ a_{m,1}\,a_{m,2}\,\cdots \,a_{m,n} \end{array}\right)\;$$

where A_*p*_,_*l*_ is a binary (decision) variable that takes a value of 1 when an indexed image point from player head detection p is related to a labeled player l; otherwise, it takes a value of 0. To locate a given player in court space, a reconstruction must be performed by considering only the points that represent the given player. The problem here is that we do not know the labels of the detected points; in other words, the associations between detected points and players are unknown. A possible solution to that problem would be to test all possible combinations, searching for the combination that minimizes the re-projection error. Unfortunately, that is a combinatorial optimization problem that can be extremely costly in terms of computation. Moreover, an additional complication is that the number of players in the scene is unknown. Thus, to estimate the assignment matrix A, we propose a constructive greedy solution that initially locates a single player. Having located this first player, the image points related to the head of the located player are dropped out of the next interaction. This heuristic drastically decreases the number of required calculations. The 3D localization of a new player stops when no more feasible solutions are available. If there are remaining player head image points that have not been assigned yet, the method takes a priori information into account (Z equal to the mean player height) to locate the last players on the court.

Let us detail our approach (Table 1) presents the notation considered herein). Basically, we seek to optimize two cost functions: (4) the minimization of the sum of the re-projection errors associated with the assigned points, which is mathematically given by the following equation:

**TABLE 1:** Notation.

*n*	Number of players
*m*	Number of points detected as player’s heads
*p*	Point index
*l*	Player label index
*x_l_k__y*_*l*_*k*__	Coordinates of player l re-projected
*w*	Total number of cameras
*k*	Camera index
*X*_*l*_*Y*_*l*_*Z*_*l*_	Court-space coordinates of player l
λ_1_*k*__,λ_2_*k*__,⋯,λ_11_*k*__	DLT parameters of camera k

(4) $$\min\,\sum_{l=1}^{n}\sum_{p=1}^{m}\left[{\left(x_{p\,k}-x_{l\,k}\right)}^{2}\,{\left(y_{p\,k}-y_{l\,k}\right)}^{2}\right]\,A_{p\,l}\;$$

and, (5) the maximization of the number of the assigned points, which is given by the following:

(5) $$\max\,\sum_{l=1}^{n}\sum_{p=1}^{m}A_{p\,l}\;$$

The rationale behind this cost function comes from the notion that greater numbers of designated image points allow for better approximations of the players’ localizations. Of course, this is not the case for outlier points, which require additional constraints to prevent their designation. Note that the cost functions expressed in Equations (4) and (5) are in conflict because a larger number of designated players increases the re-projection error. In view of this fact, we propose to merge these cost functions into a single function, as follows:

(6) $$\min\,\sum_{l=1}^{n}\left[\frac{{\left(x_{p\,k}-x_{l\,k}\right)}^{2}\,{\left(y_{p\,k}-y_{l\,k}\right)}^{2}}{{\left(\sum_{p=1}^{m}A_{p\,l}\right)}^{2}}-\sum_{p=1}^{m}A_{p\,l}\right]\,A_{p\,l}$$

**FIGURE 7:**
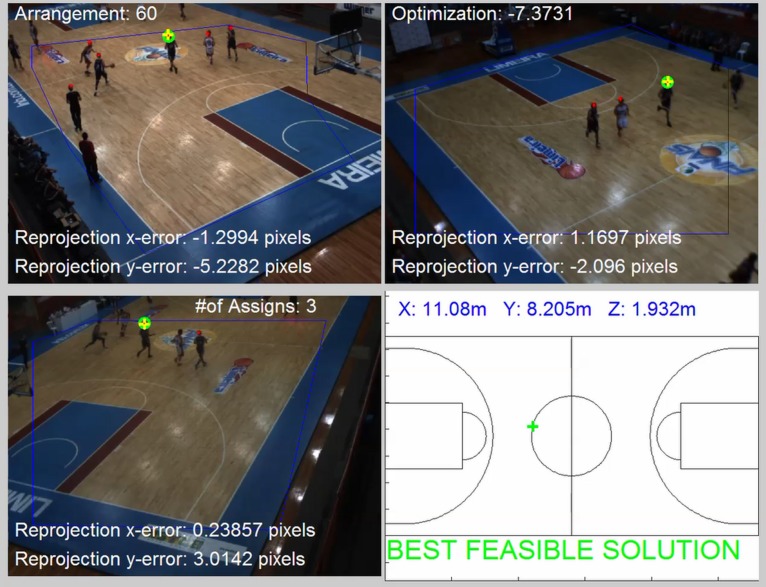
The best feasible solution obtained in first player localization. In this case, the head points are correctly assigned to the player obtaining a low re-projection error in all three cameras and then a god value for the optimization function.

**FIGURE 8:**
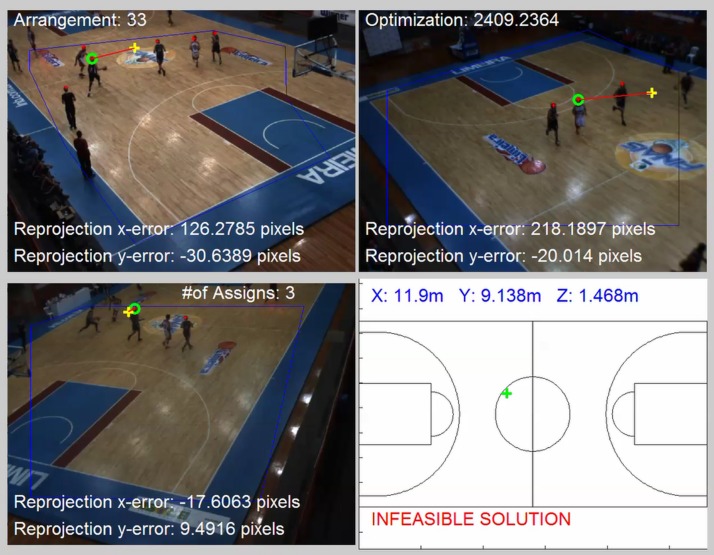
An example of combination tested with re-projection errors detailed. The head detected (red asterisk) that were designed to a given player (green circles) resulting in a poor 3D reconstruction, which the value of re-projection errors (red lines between green circles and yellow plus sign) are too large, and then provide an infeasible solution due to the one constraint.

For the greedy solution, we solve the 1st round to obtain the location of the 1st player (Figures 7, 8) and then proceed to the subsequent rounds (2nd player, 3rd player, etc.) by discarding the image points that have already been designated.

The examples can be seen in Supplementary Videos S1–S4; respectively, for first, second and third players localized in each round in straight for a given frame. The minimization of (6) must be conducted by considering the following set of constraints:

(7) $$\sum_{l=1}^{n}A_{p\,l}\leq 1,\forall p\;$$

(8) $$\sum_{\iota =1}^{m}A_{p\,\iota }\leq w,\forall l\;$$

(9) $$\sum_{\iota =1}^{n}A_{p\,\iota }\geq 2,\forall l\;$$

(10) $$A_{p\,\iota }\left[{\left(x_{p\,k}-x_{l\,k}\right)}^{2}{\left(y_{p\,k}-y_{l\,k}\right)}^{2}\right]\geq ,\forall p$$

(11) $$h_{m\,i\,n}\leq Z_{l}\leq h_{m\,a\,x}\;$$

Constraint (7) means that for every point p, only one player l can be assigned. Constraint (8) means that for every player, the number of points assigned must be equal to or less than the number of cameras. Constraint (9) means that for every player, the number of points assigned must be equal or greater than two (this is required for 3D reconstruction). Finally, Constraint (10) sets the maximum re-projection error in terms of pixel tolerance, and Constraint (11) imposes the head height limits. Having estimated the assignment matrix A, the reconstruction of the 3D position of each player can be obtained by solving a set of algebraic equations, as shown in Table 2. An example of combination tested is represented in Figure 8.

**TABLE 2:** Systems equations build upon DLT.

*p=1*	[(λ_1_*k*__−λ_3_*k*__*x*_1_*k*__)X + (λ_4_*k*__−λ_6_*k*__*x*_1_*k*__)Y + (λ_7_*k*__−λ_9_*k*__*x*_1_*k*__)Z + λ_10_*k*__−*x*_1_*k*__]*A*_1_*l*__ = 0
	[(λ_2_*k*__−λ_3_*k*__*y*_1_*k*__)X + (λ_5_*k*__−λ_6_*k*__*y*_1_*k*__)Y + (λ_8_*k*__−λ_9_*k*__*y*_1_*k*__)Z + λ_11_*k*__−*y*_1_*k*__]_*A*_1_*l*___ = 0
*p=2*	[(_λ_1_*k*___−λ_3_*k*__*x*_2_*k*__)X + (λ_4_*k*__−λ_6_*k*__*x*_2_*k*__)Y + (λ_7_*k*__−λ_9_*k*__*x*_2_*k*__)Z + λ_10_*k*__−*x*_2_*k*__]*A*_1_*l*__ = 0
	[(λ_2_*k*__−λ_3_*k*__*x*_1_*k*__)X + (λ_5_*k*__−λ_6_*k*__*y*_2_*k*__)Y + (λ_8_*k*__−λ_9_*k*__*y*_2_*k*__)Z + λ_11_*k*__−*y*_2_*k*__]*A*_1_*l*__ = 0
…	…
*p=m*	[(λ_1_*k*__−λ_3_*k*__*x*_*m*_*k*__)X + (λ_4_*k*__−λ_6_*k*__*x*_*m*_*k*__)Y + (λ_7_*k*__−λ_9_*k*__*x*_*m*_*k*__)Z + λ_10_*k*__−*x*_*m*_*k*__]*A*_*m*_*l*__ = 0
	[(λ_2_*k*__−λ_3_*k*__*x*_*m*_*k*__)X + (λ_5_*k*__−λ_6_*k*__*y*_*m*_*k*__)Y + (λ_8_*k*__−λ_9_*k*__*y*_*m*_*k*__)Z + λ_11_*k*__−*y*_*m*_*k*__]*A*_*m*_*l*__ = 0

*Direct Linear Transform (DLT) equation composing a system that solve the 3D position (X; Y; Z) for a given player.*

**FIGURE 9:**
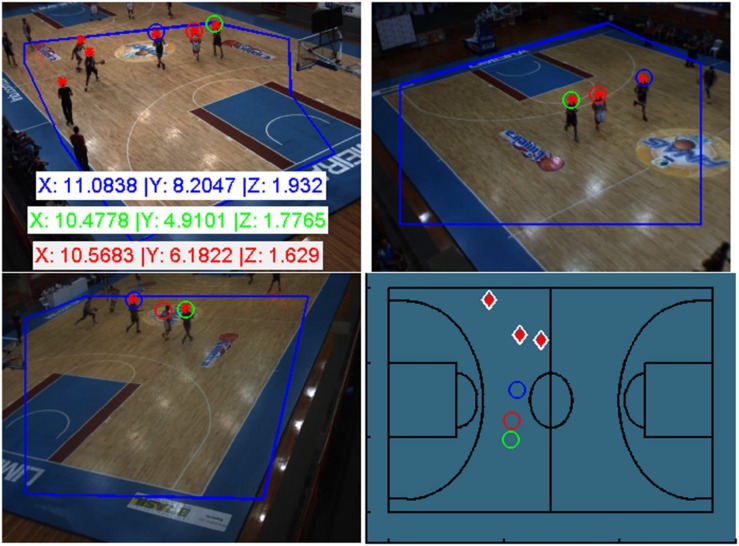
3D Localization of a given frame. Localization of 3 players, in meters, by optimization (the colored circles) and 3 remaining point. Player localizations represented on the basketball playing court **(D)**.

As already mentioned, due to the limited number of cameras along with the requirement of having at least two points from different views for 3D reconstruction, some detected points are not assigned as players and, thus, are not located in court space. In these cases, we used a priori information to solve a DLT with a fixed mean height. Figure 9 describes an example of a 3D localization result with the proposed framework; the player head detection (red asterisk) and assignments (colored circles) were obtained for a given frame as an example.

The first player localized is shown by a blue circle, the second by a green circle, and the third by a red circle. These are the top optimizations. For this example, 3 players were 3D localized by optimization and 3 players was located using a priori information (the remaining points detected only in camera 1-up left) at a position near the middle of the court (the white/red diamond). Supplementary Video S5 depicted the framework result in a short sequence movie.

## Results

For a better understanding of results presented below, the results section will be divided into 3 subsections, as named: Neural Network Classification Performance, Players Detection Evaluation and Player Localization Evaluation.

### Neural Network Classification Performance

**FIGURE 10:**
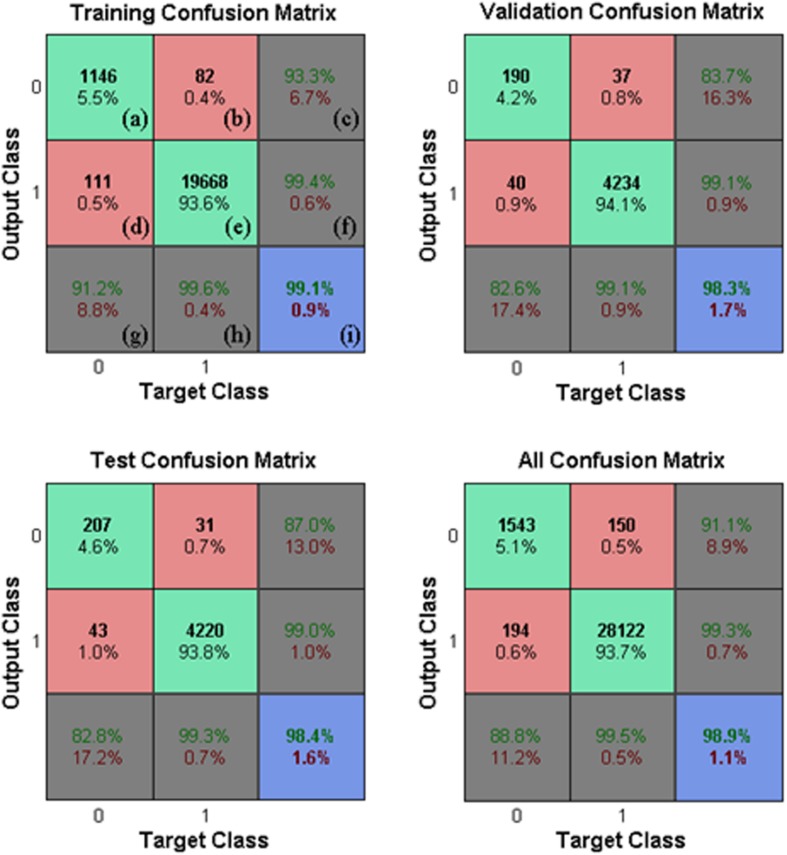
Neural Network classification. Neural network confusion matrices (Matlab confusion matrix plot model), \head” is 1 and \non-head” is 0. True classifications are denoted by green squares (a,e) and false classifications are denoted by red squares (b,d). Blue squares indicate the overall rates (correctness and error rate) of classifications (I). Gray squares (c,f,g,h) show the conditional rates (correctness and error rate) given a pre-determined target (3rd row, g,h) or given a pre-determined output (3rd column, c,f). The performance was evaluated in Game 1 for each subset.

The performance obtained by the neural network for the 30,009 samples in Game 1 is illustrated in Figure 10 using confusion matrices.

**FIGURE 11:**
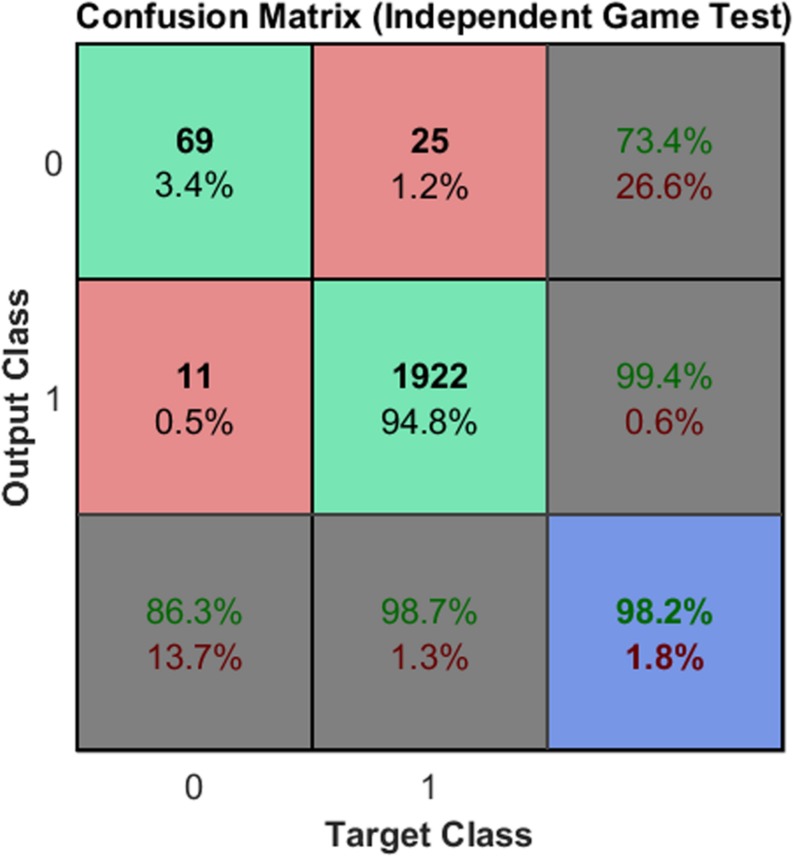
Classification in another game. Reproducibility of the classification by the neural network using images from an independent game.

Each confusion matrix depicts the occurrences of true classification (\head” classified as \head” or \non-head” classified as \non-head”), false positive classification (\non-head” classified as \head”), and false negative classification (\head” classified as \non-head”) for one of the subsets or for all subsets together. A classification was considered positive only if the head appears centralized in the square region where the HOG features were computed (e.g., in N3 in Figure 5, the head is not centralized). Finally, it is worth mentioning that the values presented in Figure 10 represent the rates of the neural network classification task, which do not correspond to the rates at the player detection stage. Figure 11 shows the results obtained for Game 2 (2,027 samples). We denote good performance, however, we cannot forget to state that is an unbalanced classification problem.

Note that – despite the fact that the neural network was trained using samples from Game – the results obtained for Game 2 were also satisfactory. Finally, in the Table 3 is presented the 2D localization error’s benchmark of our method with others presented in literature.

**TABLE 3:** Some works in the literature on player detection in team sports.

References	Sport	3D localization	Method	Localization error (2D)
Morais et al. (2014)	Futsal	No	AdaBoost detection + particle filter	~0.6 m
Needham and Boyle (2001)	Futsal	No	Background segmentation (BS)	~1.16 m
Pers et al. (2001)	Handball	No	Background segmentation + template matching	~0.32 m
Barros et al. (2011)	Handball	No	AdaBoost detection + graph	–
Alahi et al. (2009)	Basketball	No	Planar homography	–
Delannay et al. (2009)	Basketball	No	Mean-shift segmentation	–
Figueroa et al. (2006a)	Soccer	No	Background segmentation	–
Ours	Basketball	Yes	BS + neural network + combinatorial optimization	~0.16 m

*Note that not all works report a value for the player position error.*

The player detection accuracy was compared against a manual measurement (ground truth) performed using the DVideo (Campinas, SP, BRAZIL) system (Figueroa et al., 2006b; Barros et al., 2007) run by an expert operator (5 years of experience). The detection rates for each camera were calculated by considering that a detection was true when the pixel distance between a detected player’s head and the ground truth was less than 25 pixels. A pixel distance greater than 25 pixels was considered to be a false positive detection. A misdetection occurred when no point was found near a manual measure. The measures were performed only inside of a designated interest area (a pre-determined polygon). In addition, the player localization performance of the proposed framework was evaluated by comparing the real distance in meters between the player’s position as reconstructed by the proposed framework and the expert’s manual measurement in DVideo.

### Player Detection Evaluation

The results of 10,164 detections were as follows: for cameras 1, 2, and 3, respectively, the true detection rates were 78.9, 68.9, and 79.8%; the false positive rates were 2, 1.2, and 5%; and the misdetection rates were 19.1, 29.9, and 19.7%. For just the true detections, the root mean squared error (RMSE) found was 6.59 pixels.

### Player Localization Evaluation

**FIGURE 12:**
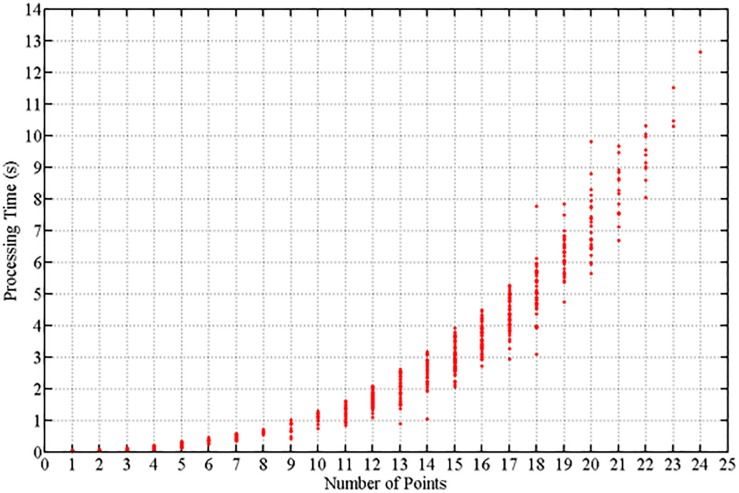
Processing time. Processing time (s) to solve the 3D localization of one player based on the number of total points detected in the camera images.

Error computation of player localization by optimization were accounted for only the players present in at least two cameras visualization inside of interest area (2,941 samples). The RMSE of 0.16 m in plane court (axis X and Y), and RMSE of 0.18 m in space court reference (axis X, Y, and Z). For the remaining (917 samples) point issues that were not assigned (not localized by optimization), the errors are shown an RMSE of 0.30 m in plane court (axis X and Y) and 0.33 m in space court (axis X, Y, and Z). The processing time required to assign the players’ locations by combinatorial optimization grows exponentially according to the number of player heads detected. The computation time required was computed for the preliminary Matlab code (not parallelized) and is depicted in Figure 12. This is the time required to measure the 3D localization of just one player, however, the localization of the next player in the optimization problem requires at least two fewer points.

## Discussion

The framework presented in the previous section and its performance are discussed in this section in more detail. The steps of player detection and localization are inherent to video-based tracking methods and play an important role in tracking. Therefore, we will discuss how tracking methods reported both in the literature and our proposal address player detection and localization and how other methods determine the players’ positions compared with our approach. Systems for data acquisition in sports must be feasible and reliable; therefore, a complete and automatic solution to measuring the players’ positions on the basketball court cannot be achieved using the knowledge from only a single research field. Thus, it was necessary to integrate tools from different fields for our approach to successfully localize the players. Searching for high-interest objects in player tracking works by integrating methods from several fields. We observed that such integrations corroborate the choice of going beyond the frontiers of knowledge in any one field. Some integration examples in the literature include image processing mixed with graph representations (Figueroa et al., 2006b), AdaBoost detection mixed with a particle filter (Lu et al., 2009; Morais et al., 2014), AdaBoost detection mixed with graph representations (Barros et al., 2011), and image processing mixed with clustering (Chen et al., 2012). Mixing together techniques from image processing, machine learning, computer vision and optimization was vital for the localization of multiple basketball players on a court. Even after the technical procedures were in place, we still faced innumerable difficult tasks in basketball player localization such as our chosen approach to seek head patterns with a Circle Hough transform, or adopting neural network classification to reject non-head points – or even using optimization to select the best assignments. State-of-the-art video-based methods for detection and player tracking in team sports take 2D positions into account, however, in the context of basketball analysis, kinematic variables that consider the vertical component of player position are essential because many sort of measurements that could be get from 3D positional data, such as specific efforts made at jump actions, fatigue index by detecting jump’s height variation during game, height of ball during passes, balls trajectory, rebounds height efficiency reached by players and vertical components of shots in different game contexts (free throws at specific moments of the match, differences of 2-point and 3-point shots, difference performance at open shots and tight shots, etc.). All these information could be useful for a more accurately diagnostics by coach’s staff in order to improve specific trainings and get a better performance from players in court. Therefore, our principal contribution lies in considering the 3D position of a given player reference point. Instead of using the bottom center of a player’s bounding box or silhouette, which attempts to represent the position of a player’s feet, we chose the player’s head as the reference point, and that decision plays a key role in our framework. There were two reasons behind this choice. First the goal was to perform a 3D reconstruction using a point that lies in court space (Z 6 = 0) and second, the solution needed to address the frequent player occlusions. Given these goals, analyzing the player’s head position was more stable and more robust to occlusions and other effects from illumination across the court. Our proposed framework comprises two main parts: (i) the detection of the players’ heads from the camera images and, (ii) the 3D reconstruction of the players’ positions. Starting with the first part, detection, we will present the levels of performance accuracy found in the literature. Then, the second part investigates the accuracy of these methods in estimating the players’ positions. The player detection rate of our proposed framework was ~71%. In other works that concentrated on indoor team sports, we found the following detection rates: 74% in a study of handball when applied to a game other than the training game (Barros et al., 2011), 70.5% in a basketball study (Delannay et al., 2009). Still focusing on basketball, the performance reported by a detection approach that used a mixed network of planar and omnidirectional cameras achieved a recall of 0.76 and a precision of 0.72 (Alahi et al., 2009). Works on outdoor team sports have also evaluated player detection rates. Experimental results from a soccer study reported 81.50 and 78.03% detection rates by two player detection methods based on a neural network and on Viola and Jones’ AdaBoost, respectively (Lehuger et al., 2007). A method for automatically tracking soccer players can locate players in 94% of video frames (Barros et al., 2007). These studies, which focused on automatic detection and tracking of outdoor team sports, used several approaches that have also been studied for indoor applications – although, for indoor sports, the camera setup (quantity, resolution, view point) and the problems faced are slightly different and include the number of players to be detected, interference from environmental features, and the spatial organization of players. Thus, outdoor studies do not allow a direct comparison with our results in detecting basketball players indoors. The median error of nearly 10 pixels in determining the players’ head positions 318 seems to be appropriate because we used images with a resolution of 1,038 × 776 pixels. An average RMS error value of ~3.4 pixels was found in a work targeted toward indoor sports applications (handball and basketball) using images with a resolution of 348 × 288 pixels obtained from gym ceiling cameras (Kristan et al., 2009). The average error in determining the position of hockey players’ foot positions from images was 20% of the width of the ground-truth box, however, this work did not present the error in terms of pixels (Lu et al., 2009). Proceeding with the discussion of the accuracy in determining a player’s position on court, even with a limited number of cameras (three in this study), it was possible to use the proposed framework to detect and localize multiple basketball players in 3D space. The median 3D error of 0.25 m was suitable considering the players were being localized on one-half of the full basketball court (14 × 15 m). Moreover, changes in the values of the parameters for optimization constraints and adding additional cameras can decrease the errors. To understand the error results through comparisons with other studies, the average cumulative error of 0.60 m in a 2D trajectory approach presented by Morais et al. (2014) used multiple-camera methodology developed for Futsal (on a playing surface of 20 × 40 m) with the errors attenuated by Fusion AdaBoost (Viola and Jones, 2001), detection from four camera images, and player appearance models. A mean error value of 0.20 m, which is associated with the uncertainties of the position of points on the visible court and not to player position error, was reported for a handball tracking study (Barros et al., 2011). Experiments showed an RMS error in player position of 0.28 m near the optical axis and 0.36 m for the court boundary when tracking handball players using ceiling cameras (Pers et al., 2001). An automatic tracking soccer study reported a spatial resolution of 0.3 m (Barros et al., 2007). A study focused on automatically tracking the positions of indoor 5-a-side football players (on a playing surface of 18 × 32 m) achieved an RMS of 1.16 m and a modal value below 40 cm compared with manual tracking (Needham and Boyle, 2001). In our approach, we attack the problem of localizing multiple basketball players using a video-based framework. Yet another alternative for tracking player position is to use a global positioning system (GPS), however, at present, the errors from GPS measurements are too large even outdoors (Gray et al., 2010), and indoor use is impracticable. To provide an example of GPS accuracy, ~50% of the GPS coordinates were within 2.5 m in a static position test (Mohamad et al., 2009). However, GPS systems often do not work at all in basketball gyms. In addition, the rules of many sports do not allow the players to use GPS devices. Because no temporal information was used in our proposed framework, the results could be improved by using the players’ trajectories to help predict their current positions, filtering the player trajectory data to discard outlier positions. Although linking temporal information to player detection was not the goal of this study, it is an aspect that could be investigated in future studies.

## Conclusion

A video-based framework for automatic 3D localization of multiple basketball players was described in the context of official games. Player detection was based on image processing techniques and – considering the complexity of basketball games – the classification problem presented satisfactory results. The classification procedure was essential to properly reject head candidate points (for example, to reject other body parts such as a raised arm).

A combinatorial optimization problem was solved with a greedy heuristic and provided satisfactory results in accurately determining both the number of players in a scene and their positions. Knowing the player’s positions in 3D relative to the court is crucial for basketball performance analysis due to the nature of the sport. This work helps to further systems development aiming to acquire 3D player position data during competitions, and the application can be extended to other indoor team sports in which a vertical component is relevant.

## Data Availability Statement

All the datasets generated and analyzed for this study are included in the article/Supplementary Material.

## Ethics Statement

Ethical review and approval was not required for the study on human participants in accordance with the local legislation and institutional requirements. The patients/participants provided their written informed consent to participate in this study. Written informed consent was obtained from the individual(s) for the publication of any potentially identifiable images or data included in this article.

## Author Contributions

LMo, LD, and MM: writing-original draft preparation. LMo, LD, MM, AJ, and LMe: investigation, data analysis, and conceptualization. LMo, LD, MM, and LMe: funding acquisition.

## Conflict of Interest

The authors declare that the research was conducted in the absence of any commercial or financial relationships that could be construed as a potential conflict of interest.

## Supplementary Material

VIDEO S1 Player detection process. Candidate points obtained through some image processing including a background segmentation approach to be classificated into head or non-head.

VIDEO S2 First round of optimization. Test of all possible combinations for first player localization with the best feseable solution. Head points detected depicted as red asterisk, points assign to the player showed as green circles, camera reprojection of 3D reconstructed player in the current combination tested marked as yellow plus sign, and, court plane representation with 3D player position reconstructed for tested solution depicted as green plus sign.

VIDEO S3 Second round of optimization. Test for all combinations for localization of the second player remember that head point assigned to the first player are excluded for current cominations. Head points detected depicted as red asterisk, points assign to the player showed as green circles, camera reprojection of 3D reconstructed player in the current combination tested marked as yellow plus sign, and, court plane representation with 3D player position reconstructed for tested solution depicted as green plus sign.

VIDEO S4 Third round of optimization. Test for all combinations for localization of the second player remember that head point assigned to the first player are excluded for current cominations. Head points detected depicted as red asterisk, points assign to the player showed as green circles, camera reprojection of 3D reconstructed player in the current combination tested marked as yellow plus sign, and, court plane representation with 3D player position reconstructed for tested solution depicted as green plus sign.

VIDEO S5 Player Localization in game. A sequence example of player localization. The player localized in 3D court spacare reprojected in each camera keeping the same color circles for the same player.
